# Pain- and depression-related regional homogeneity changes in ankylosing spondylitis: a functional magnetic resonance imaging study

**DOI:** 10.3389/fneur.2025.1521531

**Published:** 2025-06-04

**Authors:** Kelei Hua, Peijun Wang, Bin Xia, Haiying Wang, Tianyue Wang, Jin Fang, Yi Yin, Yike Tu, Guihua Jiang

**Affiliations:** ^1^Department of Medical Imaging, The Affiliated Guangdong Second Provincial General Hospital of Jinan University, Guangzhou, China; ^2^Department of Medical Imaging, Southern Medical University Hospital of Integrated Traditional Chinese and Western Medicine, Guangzhou, China; ^3^Guangzhou Key Laboratory of Molecular Functional Imaging and Artificial Intelligence for Major Brain Diseases, The Affiliated Guangdong Second Provincial General Hospital of Jinan University, Guangzhou, China

**Keywords:** ankylosing spondylitis, pain, depression, fMRI, regional homogeneity

## Abstract

**Purpose:**

Because of their ongoing pain, patients with ankylosing spondylitis (AS) are more likely to experience depression. Effective treatment remains a challenge. Additionally, the mechanisms of and relationships between AS-related pain and depression are inadequately understood. This study explored the regional homogeneity (ReHo) alterations linked to pain and depression in patients with AS.

**Methods:**

In total, 43 patients with AS (40 men, 3 women) and 46 controls who were matched by age and sex were recruited. The patients were clinically assessed based on Bath Ankylosing Spondylitis Disease Activity Index, the Total Back Pain (TBP) and Hamilton Rating Scale for Depression (HAMD) scores, erythrocyte sedimentation rate, and high-sensitivity C-reactive protein level. The ReHo differences based on 3-T magnetic resonance imaging were compared between patients with and without AS. Associations between significant variables and pain and depression were further explored.

**Results:**

Patients with AS had decreased ReHo values within the left superior temporal gyrus and right paracentral lobule and increased values within the left precuneus and right middle frontal gyrus compared to healthy controls (*p* < 0.05, FDR correction). The left precuneus ReHo value negatively correlated with the TBP and HAMD scores. The right paracentral lobule ReHo value positively correlated with the AS duration and TBP score. The left precuneus had increased neural activity in patients with AS, which may lead to abnormal sensory responses, issues in emotion regulation, and deviations in information processing.

**Conclusion:**

This work provides fresh understanding of the brain processes behind depression and pain associated with AS. Stratifying patients based on features with significant correlations with pain and depression could help identify those at risk and thus apply individualized treatment.

## 1 Introduction

Low back discomfort is a hallmark of ankylosing spondylitis (AS), an immune-mediated systemic inflammatory illness that primarily affects the spine and sacroiliac joints (1). AS predominantly affects young adults, with symptoms typically appearing in late adolescence or early adulthood and has a male predominance. However, the proportion of AS patients over the age of 45 is estimated to occur in around 3.5–13.8% of all cases of AS (2). Peripheral origins of AS pain have been linked to local inflammation (3). However, recent research has shown that central pain mechanisms play a crucial role in AS (4–6). Pain is also an acknowledged primary outcome for evaluating treatment responsiveness in AS trials. Because of their persistent pain, patients with AS are also more likely to experience mental health issues (7). People who have chronic pain often have depressive symptoms, which have been observed in 51% of patients with AS (8). Additionally, compared to individuals who merely experience chronic pain, those who also have psychological comorbidities like depression are likely to have a worse outcome (9–11).

In people with chronic pain, research conducted in the last few decades has shown a reciprocal association between pain and depression. A longitudinal study by Kroenke et al. (12) demonstrated that pain was a strong predictor of future depression severity, and that depression was substantially linked to future pain intensity prediction. The brain mechanisms that underlie the mutual relationship between pain and depression have since been examined using neuroimaging techniques (13–15). The importance is found in the same brain plasticity that leads to the development and progression of depression and chronic pain at the same time. However, no research has examined how AS affects the central nervous system in relation to both pain and depression.

To investigate the functional alterations in the brains of patients with AS, this study combined psychophysical and functional magnetic resonance imaging (fMRI) methods. Using the Hamilton Rating Scale for Depression (HAMD), we further investigated the relationships between these brain changes and the length of pain and emotional comorbidities associated with pain. Overall, we aimed to improve our knowledge of the pathophysiological mechanisms of AS and establish a robust foundation for patient diagnosis based on mechanisms, which could propel a shift toward personalized therapy.

## 2 Materials and methods

### 2.1 Participants

The Ethics Committee of The Affiliated Guangdong Second Provincial General Hospital of Jinan University approved this study. Each participant signed an informed consent form after being fully informed about the study. The following criteria were met in order to recruit patients with AS: active AS diagnosis aligning with the modified New York criteria (16), the use of non-steroidal anti-inflammatory drugs only at a stable dose in the event of pain, the avoidance of biological agents during the study or at any other time, and an average Total Back Pain (TBP) score of ≥3 (on a 10-point scale, where 0 represents no pain and 10 represents the worst pain imaginable) during the previous week. Suitability for inclusion was not assessed using the depression severity score. Age- and sex-matched healthy controls (HCs) were also included. The basic inclusion criteria for all study participants were as follows: age 16–50 years, no previous neurological disease diagnosis, no major surgery within the last 2 years, and no additional MRI contraindications.

### 2.2 Clinical assessments

The TBP and BASDAI scores were part of the clinical evaluation of every AS patient. BASDAI is a commonly used disease activity index in AS (17). A trained rheumatologist performed the BASDAI assessments. Spinal pain, joint pain/swelling, exhaustion, localized discomfort, and morning stiffness are all included in the BASDAI score, which offers a thorough overview of symptom severity on a scale of 0 to 10 (10 = the highest disease severity). A week prior to their fMRI scans, patients with AS and HCs were assessed using the HAMD scale.

### 2.3 MRI scans

A 3.0-T MRI scanner (Ingenia; Philips, Best, Netherlands) was used in the Department of Medical Imaging at The Affiliated Guangdong Second Provincial General Hospital of Jinan University to acquire MRI data for every subject. High-resolution anatomical scanning and resting-state fMRI (rs-fMRI) were performed on each participant. With the following sequence settings, an echo-planar imaging sequence was used to obtain the rs-fMRI data: repetition time (TR) = 2000 ms; echo time (TE) = 30 ms; flip angle = 90°; field of view = 224 × 224 mm^2^; resolution = 64 × 64 matrix; number of slices = 33; slice thickness = 3.5 mm with a 0.7 mm gap; total volumes = 240; and acquisition time ≈ 8 min. To assess each participant’s degree of cooperation throughout the scan, questions about whether they dozed off and opened their eyes were asked both during and after the MRI. Participants who could not follow the instructions were rescanned to acquire new rs-fMRI information.

The following settings were used to obtain high-resolution anatomical images: TR = 7.9 ms; TE = 3.7 ms; flip angle = 8°; acquisition matrix = 256 × 256; field of view = 256 × 256 mm^2^; slice thickness = 1.0 mm; and 185 sagittal slices without gaps, covering the whole brain.

### 2.4 Data processing

The data were preprocessed using the Statistical Parametric Mapping (SPM12) and Data Processing and Analysis for Brain Imaging (DPABI) toolboxes running on MATLAB 2014b (MathWorks, Natick, MA) (18). The process included the following steps. First, the original images were converted into NIFTI format. Second, the first ten volumes were discarded to exclude the influence of machine signal instability and the participant’s adaptation process on the results. Third, the difference in the time between the acquired images of each slice was corrected. Fourth, images from participants with head movement >1.5 mm and rotation >1.5° were realigned to reduce the influence of head movement noise on the signal. Fifth, images were normalized to the standard echoplanar imaging template, and each voxel was resampled to 3 × 3 × 3 mm^3^. Sixth, functional and anatomical images (T1-weighted images) were registered to accurately locate the functional activation area. Finally, the data were detrended and filtered (0.01–0.08 Hz); both high-frequency physiological noise (such as breathing, heartbeat, etc.) and low-frequency linear drift had less of an impact.

### 2.5 Regional homogeneity

Regional homogeneity (ReHo) calculations were executed as previously reported (19). Briefly, Kendall’s coefficient of concordance (KCC) of a given voxel time series with the nearest 26 adjacent voxels was used to estimate ReHo on a voxel-by-voxel basis. Each participant received a unique KCC map after the KCC value was computed as a voxel. An 8-mm full-width at half-maximum Gaussian kernel was used to spatially smooth the data to minimize noise and residuals in the gyrus anatomy.

### 2.6 Statistical analyses

A two-sample t-test was used to evaluate significant differences in age and education between the AS and HC groups, and chi-squared tests were used to analyze differences depending on sex. SPSS version 20.0 (IBM, Armonk, NY) was used for statistical analyses. The ReHo values of patients with AS and HCs were compared using two-sample t-tests. *p*-values of less than 0.05 were regarded as statistically significant when adjusted for false discovery rate. Clinical outcomes (BASDAI, TBP, and HAMD scores, serum C-reactive protein [CRP] level, erythrocyte sedimentation rate [ESR], and disease duration) were analyzed for AS patients, as were partial correlations between the ReHo values of various brain regions that revealed group differences. Significant p-values were defined as less than 0.05.

## 3 Results

### 3.1 Participant demographics

We enrolled 46 HCs and 43 patients with AS. However, because of significant head movement during imaging, one patient and one HC were eliminated. Therefore, 45 HCs and 42 patients were ultimately kept. Table 1 provided specifics on the sample’s characteristics. There was no difference between the two groups in terms of years of schooling, sex, or age (*p* < 0.05). Furthermore, the patient group’s HAMD scores were greater.

**Table 1:** Demographic characteristics of the patients with AS and the healthy controls (HC).

Characteristics	AS (*n* = 43)	HC (*n* = 46)	*p*-value
Age (years)	25.07 ± 6.81	25.54 ± 6.29	0.734^b^
Gender (male/female)	40/3	42/4	0.763^a^
Education (years)	11.93 ± 2.68	11.89 ± 3.08	0.950^b^
Total back pain	5.98 ± 1.29	N/A	–
AS duration (years)	7.12 ± 5.15	N/A	–
BASDAI	4.85 ± 1.36	N/A	–
HAMD	24.44 ± 5.62	N/A	–
ESR	17.24 ± 9.17	N/A	–
CRP	11.64 ± 9.32	N/A	–

Data were expressed as mean ± SD. BASDAI, Bath Ankylosing Spondylitis Disease Activity Index; ESR, erythrocyte sedimentation rate; CRP, C-reactive protein.

a *p*-value was obtained using the two-tailed Chi-squared test.

b *p*-value was obtained using the two-sample, two-tailed *t*-test.

### 3.2 Differences in ReHo between two groups

**Figure 1:**
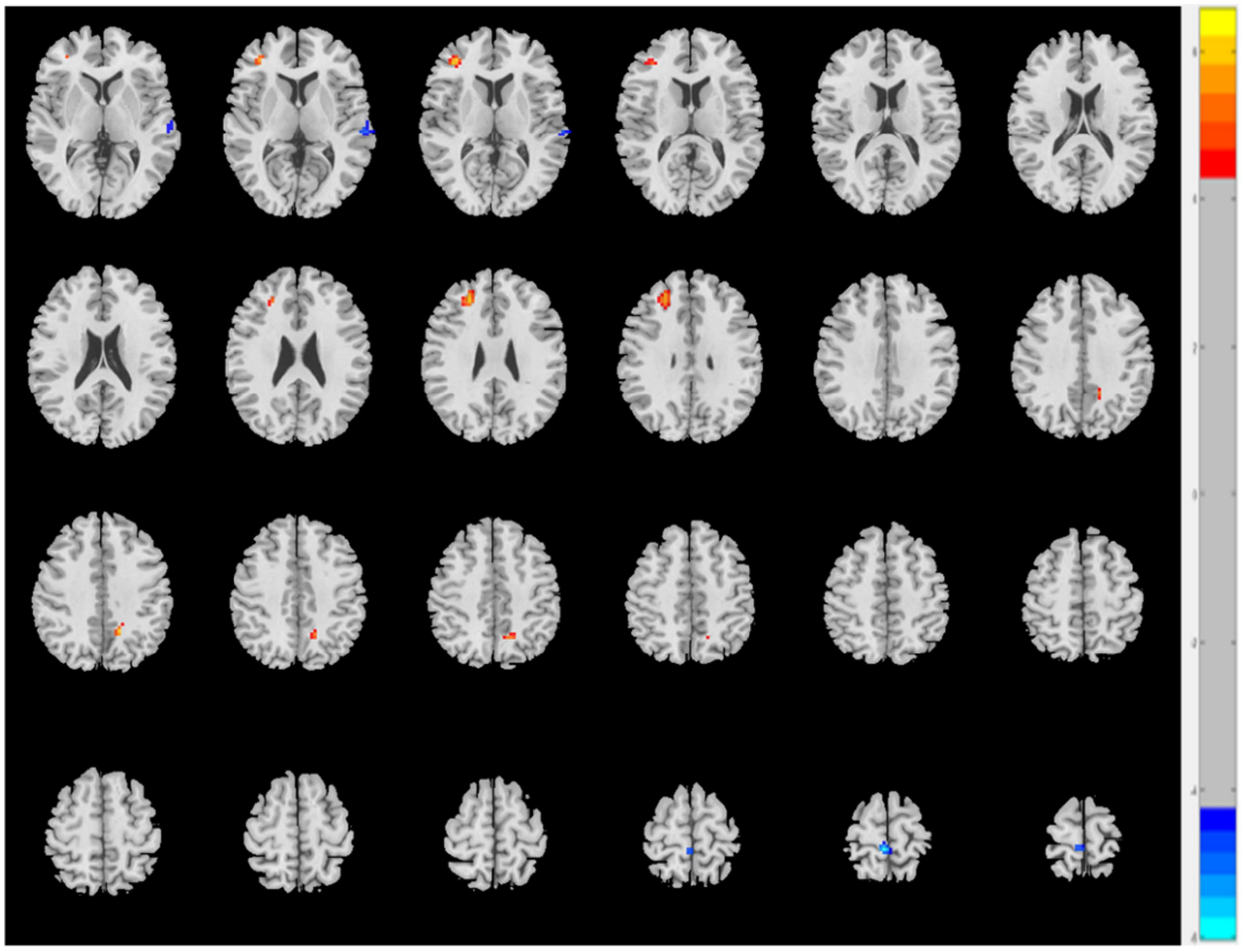
The AS group shows increased ReHo values in the left precuneus and right middle frontal gyrus and decreased ReHo values in the left superior temporal gyrus and right paracentral lobule relative to the HC (*p* < 0.05, FDR correction).

The AS group displayed decreased ReHo values in the left superior temporal gyrus and right paracentral lobule and increased ReHo values in the left precuneus and right middle frontal gyrus (MFG) when compared to the HC group (*p* < 0.05, FDR correction) (Table 2 and Figure 1).

**Table 2:** Brain region of Reho differences between AS group and HC group.

**Brain area**	**MNI**				
	**Voxel size**	**x**	**y**	**z**	***T* value**
Frontal_Mid_R	28	24	36	27	6.180
Temporal_Sup_L	22	−63	−24	6	−4.849
Precuneus_L	20	−15	−51	39	6.002
Paracentral_Lobule_R	16	3	−36	69	−6.056

### 3.3 Correlation analysis

**Figure 2:**
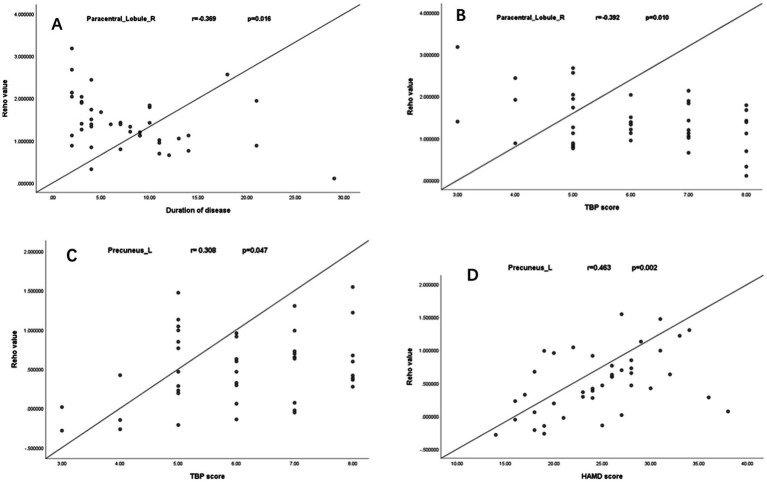
Negative correlation between ReHo values of right paracentral lobule with the duration of AS and TBP scores **(A,B)**; Positive correlation between the ReHo values of the left precuneus with TBP and HAMD scores **(C,D)**.

The right paracentral lobule’s ReHo values showed a negative correlation with the duration of AS and TBP scores (Figures 2A,B), while the left precuneus’ ReHo values showed a positive correlation with both TBP and HAMD scores (Figures 2C,D). The TBP, HAMD score, ESR, and CRP did not significantly correlate with changes in ReHo values in the left superior temporal gyrus or right MFG.

## 4 Discussion

This study explored pain- and depression-related ReHo changes in patients with AS and the association between altered ReHo and clinical features, such as TBP, the HAMD score, ESR, and CRP level. Compared to HCs, patients with AS showed considerably decreased ReHo values in the right paracentral lobule and left superior temporal gyrus and significantly increased ReHo values in the left precuneus and right MFG while at rest. Furthermore, the TBP and HAMD scores positively correlated with the left precuneus ReHo values, while the AS duration and TBP score negatively correlated with the right paracentral lobule ReHo values. Correlations between ReHo value changes in the right MFG and left superior temporal gyrus and TBP, the HAMD score, ESR, and CRP were not identified.

Neural activity in the default mode network (DMN) is inhibited when the brain performs external activities but is highly active during the resting state. The DMN is essential for self-reflection, memory, and attention (20). However, in the present study, patients with AS showed increased ReHo values in the left precuneus and decreased ReHo values in the left superior temporal gyrus, which are two are important DMN structures (21).

The precuneus plays a crucial role in cognitive function networks and is a key part of the medial pain system, which is primarily in charge of processing unpleasant emotions brought on by pain or other discomforts (22). According to studies, individuals who suffer from migraines without aura exhibit a substantial rise in bilateral precuneus ReHo values and increased functional connectivity in the left precuneus within the DMN (23). Goffaux et al. (24) found that pain-induced responses in the contralateral precuneus of healthy adults are closely related to pain sensitivity, and Emerson et al. (25) reported that there is a substantial negative correlation between pain sensitivity and grey matter density in the bilateral posterior cingulate cortex, precuneus, intraparietal sulcus, and inferior parietal lobule areas. However, precuneus abnormalities have been reported in recent functional and structural MRI studies on major depressive disorder (26, 27). Furthermore, a poorer connection between the precuneus and the subcallosal anterior cingulate was substantially and exclusively linked to higher depression severity scores in patients with chronic depression (26). Another study suggested that the precuneus’ structural-functional connection offers key components that can be used to model several mental illnesses, including depression (28). Additionally, the precuneus is a medial parietal region that is directly related to memory, navigation, and spatial function and is essential for DMN activity and cognitive processing. In addition, the TBP and HAMD scores positively correlated with the left precuneus ReHo values. Thus, it has been proposed that sensory impairment, abnormal information processing, and deficiencies in emotion regulation may result from increased neuronal activity in the left precuneus in patients with AS. This could cause significant changes in the DMN before sensory impairment and depressive episodes appear. While the current findings provide preliminary support for the observed associations, their robustness and generalizability must be rigorously evaluated through large-scale, multicenter prospective studies employing standardized protocols.

Patients with AS exhibited enhanced spontaneous neuronal activity in the right MFG in the frontal lobe. Changes in the MFG’s functional activity may result in incorrect reactions to emotional events, as the MFG aids in regulating the intensity of reactions to emotional stimuli. In terms of cognitive control and emotional regulation processes, particularly those pertaining to enjoyment, the dorsolateral prefrontal cortex (DLPFC) is typically regarded as a fundamental brain region (29, 30). These elements affect the symptoms of depression. Notably, depression has been closely associated with the DLPFC, of which the MFG is an essential component. fMRI investigations of major depressive disorder have often shown hyperactivity in the DLPFC and higher functional activity in this area of the brain (31), and hyperactivity in this brain region has been correlated with depression severity (32). Additionally, the DLPFC is the primary region that repetitive transcranial magnetic stimulation targets to treat depression (33). The ReHo results in this study further demonstrate that AS pain combined with depression may induce MFG dysfunction. The DLPFC is often activated during pain neuroimaging. It should be noted that while it is not the only active region, it might be a crucial node in networks linked to pain modulation and nociceptive processing (34). However, their function in pain is still unknown. There were no correlations found between the clinical features and the MFG ReHo values. We postulated that these modifications might be adaptive/self-regulating processes involving the attention and somatosensory networks. To comprehensively validate the hypothesized adaptive interplay between attentional and somatosensory networks, future investigations must integrate closed-loop neurofeedback systems, multimodal imaging protocols, and longitudinal intervention designs.

The superior temporal gyrus is the primary system for processing auditory information (35). Changes in the function of the somatosensory brain areas are the main cause of pain, which is a complex psychophysiological phenomenon (36). However, according to some research, pain may also have an impact on the visual and auditory networks (37, 38). In a study of individuals with persistent musculoskeletal pain, Coppieters et al. (39) discovered a correlation between reduced regional gray matter volume in the superior frontal and temporal gyri and increased pain intensity and pressure pain sensitivity. Clarifying the pathophysiology and etiology of AS can be achieved by comprehending the function of the auditory network. In this study, the local consistency in the left superior temporal gyrus was reduced, possibly due to the excessive attention paid by patients with chronic pain to their pain, which in turn affected other body sensory perception systems. Another possible aberrant neurological mechanism of chronic pain that needs more investigation is excessive attention to pain.

The paracentral lobule is crucial for somatosensation and regulates motor and sensory innervation (40). The somatosensory cortex, a sensory/motivational association region implicated in the affective/discriminative components of pain, is part of the human brain’s widely dispersed pain pathways, according to numerous studies (41–43). Furthermore, neuroscientific studies show that alterations in somatosensory regions—afferent nociceptive brain regions—occur in tandem with the effects of expectation on the experience of subjective pain. Descending pain modulatory circuits also have a role in mediating this impact (44). The primary and secondary somatosensory, anterior cingulate, insular, and thalamic regions that are active when experiencing severe pain were assessed by a meta-analysis of positron emission tomography, fMRI, electroencephalogram, and magnetoencephalography studies. These regions were examined as the basic human nociceptive processing’s central network (36). AS is a type of inflammatory arthritis that carries a considerable mental health burden; the risk of depression is 51% higher in patients with AS than in those without AS (7). In this study, decreased ReHo was observed in the right paracentral lobule, and the right paracentral lobule ReHo values negatively correlated with the AS duration and TBP score. Our findings indicate that pain-associated neural networks exhibit extensive crosstalk and dynamically dysregulate the formation and maintenance of synchronized oscillatory activity within sensory-cognitive integration hubs. This dysregulation potentially underpins the maladaptive plasticity observed in chronic pain states. Future studies employing closed-loop neuromodulation could test whether restoring inter-network coherence reverses these pathophysiological signatures.

There are various limitations to our investigation. First, we used a cross-sectional study design. Consequently, we are unable to determine the cause of the identified anomalies in the brain. Alternatively, the changes we detected might be the downstream signals. Nevertheless, these findings are still significant because, currently, an accepted objective measure of pain and depression for AS does not exist. Nonetheless, it is crucial to confirm our findings with the same cohort using a longitudinal strategy. Second, it remains unclear whether the correlations we found for pain and depression were generic or specific to AS, because there are few similarly constructed studies in this area. Therefore, additional validation of distinct pain and depression disorders is required. Third, the single-center design of this study, coupled with its moderate sample size, may have reduced statistical power and compromised the generalizability of findings to broader populations. To enhance methodological rigor, future investigations should prioritize multicenter collaboration with standardized protocols, which would not only increase sample size but also enable validation of current results across diverse clinical settings and heterogeneous patient cohorts. Fourth, this study lacked stratification by AS clinical severity or phenotypic subgroups, potentially obscuring critical heterogeneity in outcome associations across disease spectra. Future investigations should incorporate standardized disease activity metrics and machine learning-driven cluster analysis to delineate phenotype-specific outcome trajectories.

## 5 Conclusion

This preliminary study explored ReHo value changes in patients with AS compared to HCs, identifying increased and decreased ReHo values in different brain regions, some of which were consistent with the results of previous fMRI studies. Additionally, pain and depression levels were significantly correlated with functional abnormalities in the brain. These findings contribute to our knowledge of the brain underpinnings of AS and offer proof of neurological dysfunction associated with AS. Hence, further investigations into the pathophysiology of regions altered ReHo values should be performed.

## Data Availability

The original contributions presented in the study are included in the article/supplementary material, further inquiries can be directed to the corresponding author.

## References

[1] Sieper J Poddubnyy D. Axial spondyloarthritis. Lancet. (2017) 390:73–84. doi: 10.1016/S0140-6736(16)31591-4, PMID: 28110981

[2] He W Yang H Yang X Huang J Wu Z. Global research trends in biological therapy for ankylosing spondylitis: a comprehensive visualization and bibliometric study (2004-2023). Hum Vaccin Immunother. (2025) 21:2445900. doi: 10.1080/21645515.2024.2445900, PMID: 39813123 PMC11740677

[3] Braun J Sieper J. Ankylosing spondylitis. Lancet. (2007) 369:1379–90. doi: 10.1016/S0140-6736(07)60635-7, PMID: 17448825

[4] Wu Q Inman RD Davis KD. Neuropathic pain in ankylosing spondylitis: a psychophysics and brain imaging study. Arthritis Rheum. (2013) 65:1494–503. doi: 10.1002/art.37920, PMID: 23460087

[5] Borman P Kaygisiz F Yaman A. Neuropathic component of low back pain in patients with ankylosing spondylitis. Mod Rheumatol. (2021) 31:462–7. doi: 10.1080/14397595.2020.1754322, PMID: 32271113

[6] Kedyk I Stanislavchuk M. Clinical characteristics of ankylosing spondylitis patients depending on neuropathic pain. Reumatologia. (2023) 61:104–8. doi: 10.5114/reum/163223, PMID: 37223366 PMC10201386

[7] Wilson N Liu J Adamjee Q di Giorgio S Steer S Hutton J. Exploring the emotional impact of axial Spondyloarthritis: a systematic review and thematic synthesis of qualitative studies and a review of social media. BMC Rheumatol. (2023) 7:26. doi: 10.1186/s41927-023-00351-w, PMID: 37608395 PMC10464274

[8] Park JY Howren AM Zusman EZ Esdaile JM de M. The incidence of depression and anxiety in patients with ankylosing spondylitis: a systematic review and meta-analysis. BMC Rheumatol. (2020) 4:12. doi: 10.1186/s41927-019-0111-6 32159073 PMC7050143

[9] Meesters JJ Bremander A Bergman S Petersson IF Turkiewicz A Englund M. The risk for depression in patients with ankylosing spondylitis: a population-based cohort study. Arthritis Res Ther. (2014) 16:418. doi: 10.1186/s13075-014-0418-z, PMID: 25209603 PMC4180137

[10] Debeer P Commeyne O De Cupere I Tijskens D Verhaegen F Dankaerts W. The outcome of hydrodilation in frozen shoulder patients and the relationship with kinesiophobia, depression, and anxiety. J Exp Orthop. (2021) 8:85. doi: 10.1186/s40634-021-00394-3, PMID: 34591188 PMC8484410

[11] İzci Duran T Pamukçu M Ulusoy H Altınbaş K. Evaluation of the role of affective temperamental features, automatic thoughts, and symptom interpretation on disease activity in patients with axial spondyloarthritis. Alpha Psychiatry. (2023) 24:68–74. doi: 10.5152/alphapsychiatry.2023.22908, PMID: 37144054 PMC10152049

[12] Kroenke K Wu J Bair MJ Krebs EE Damush TM Tu W. Reciprocal relationship between pain and depression: a 12-month longitudinal analysis in primary care. J Pain. (2011) 12:964–73. doi: 10.1016/j.jpain.2011.03.003, PMID: 21680251 PMC3222454

[13] Doan L Manders T Wang J. Neuroplasticity underlying the comorbidity of pain and depression. Neural Plast. (2015) 2015:504691. doi: 10.1155/2015/504691, PMID: 25810926 PMC4355564

[14] Humo M Lu H Yalcin I. The molecular neurobiology of chronic pain-induced depression. Cell Tissue Res. (2019) 377:21–43. doi: 10.1007/s00441-019-03003-z, PMID: 30778732

[15] Sheng J Liu S Wang Y Cui R Zhang X. The link between depression and chronic pain: neural mechanisms in the brain. Neural Plast. (2017) 2017:9724371. doi: 10.1155/2017/9724371, PMID: 28706741 PMC5494581

[16] van der Linden S Valkenburg HA Cats A. Evaluation of diagnostic criteria for ankylosing spondylitis. A proposal for modification of the New York criteria. Arthritis Rheum. (1984) 27:361–8. doi: 10.1002/art.1780270401, PMID: 6231933

[17] Garrett S Jenkinson T Kennedy LG Whitelock H Gaisford P Calin A. A new approach to defining disease status in ankylosing spondylitis: the Bath ankylosing spondylitis disease activity index. J Rheumatol. (1994) 21:2286–91. Available at: https://pubmed.ncbi.nlm.nih.gov/7699630/7699630

[18] Yan CG Wang XD Zuo XN Zang YF. DPABI: Data Processing & Analysis for (resting-state) brain imaging. Neuroinformatics. (2016) 14:339–51. doi: 10.1007/s12021-016-9299-4, PMID: 27075850

[19] Zang Y Jiang T Lu Y He Y Tian L. Regional homogeneity approach to fMRI data analysis. NeuroImage. (2004) 22:394–400. doi: 10.1016/j.neuroimage.2003.12.030, PMID: 15110032

[20] Menon V. 20 years of the default mode network: a review and synthesis. Neuron. (2023) 111:2469–87. doi: 10.1016/j.neuron.2023.04.023, PMID: 37167968 PMC10524518

[21] Raichle ME. The brain's default mode network. Annu Rev Neurosci. (2015) 38:433–47. doi: 10.1146/annurev-neuro-071013-014030, PMID: 25938726

[22] Cavanna AE Trimble MR. The precuneus: a review of its functional anatomy and behavioural correlates. Brain. (2006) 129:564–83. doi: 10.1093/brain/awl004, PMID: 16399806

[23] Qin ZX Su JJ He XW Zhu Q Cui YY Zhang JL. Altered resting-state functional connectivity between subregions in the thalamus and cortex in migraine without aura. Eur J Neurol. (2020) 27:2233–41. doi: 10.1111/ene.14411, PMID: 32562320

[24] Goffaux P Girard-Tremblay L Marchand S Daigle K Whittingstall K. Individual differences in pain sensitivity vary as a function of precuneus reactivity. Brain Topogr. (2014) 27:366–74. doi: 10.1007/s10548-013-0291-0, PMID: 23636269

[25] Emerson NM Zeidan F Lobanov OV Hadsel MS Martucci KT Quevedo AS. Pain sensitivity is inversely related to regional grey matter density in the brain. Pain. (2014) 155:566–73. doi: 10.1016/j.pain.2013.12.004, PMID: 24333778 PMC3944887

[26] Rubart AK Zurowski B Veer IM Schön D Göttlich M Klein JP. Precuneus connectivity and symptom severity in chronic depression^☆^. Psychiatry Res Neuroimaging. (2022) 322:111471. doi: 10.1016/j.pscychresns.2022.111471, PMID: 35378340

[27] Wang K Hu Y Yan C Li ML Wu YJ Qiu J. Brain structural abnormalities in adult major depressive disorder revealed by voxel- and source-based morphometry: evidence from the REST-meta-MDD consortium. Psychol Med. (2023) 53:3672–82. doi: 10.1017/S0033291722000320, PMID: 35166200

[28] Dadario NB Sughrue ME. The functional role of the precuneus. Brain. (2023) 146:3598–607. doi: 10.1093/brain/awad181, PMID: 37254740

[29] Niendam TA Laird AR Ray KL Dean YM Glahn DC Carter CS. Meta-analytic evidence for a superordinate cognitive control network subserving diverse executive functions. Cogn Affect Behav Neurosci. (2012) 12:241–68. doi: 10.3758/s13415-011-0083-5, PMID: 22282036 PMC3660731

[30] Frank DW Dewitt M Hudgens-Haney M Schaeffer DJ Ball BH Schwarz NF. Emotion regulation: quantitative meta-analysis of functional activation and deactivation. Neurosci Biobehav Rev. (2014) 45:202–11. doi: 10.1016/j.neubiorev.2014.06.010, PMID: 24984244

[31] Kaiser RH Andrews-Hanna JR Wager TD Pizzagalli DA. Large-scale network dysfunction in major depressive disorder: a Meta-analysis of resting-state functional connectivity. JAMA Psychiatry. (2015) 72:603–11. doi: 10.1001/jamapsychiatry.2015.0071, PMID: 25785575 PMC4456260

[32] Grimm S Beck J Schuepbach D Hell D Boesiger P Bermpohl F. Imbalance between left and right dorsolateral prefrontal cortex in major depression is linked to negative emotional judgment: an fMRI study in severe major depressive disorder. Biol Psychiatry. (2008) 63:369–76. doi: 10.1016/j.biopsych.2007.05.033, PMID: 17888408

[33] Zheng H Zhang L Li L Liu P Gao J Liu X. High-frequency rTMS treatment increases left prefrontal myo-inositol in young patients with treatment-resistant depression. Prog Neuro-Psychopharmacol Biol Psychiatry. (2010) 34:1189–95. doi: 10.1016/j.pnpbp.2010.06.009, PMID: 20600472

[34] Seminowicz DA Moayedi M. The dorsolateral prefrontal cortex in acute and chronic pain. J Pain. (2017) 18:1027–35. doi: 10.1016/j.jpain.2017.03.008, PMID: 28400293 PMC5581265

[35] Petrides M. On the evolution of polysensory superior temporal sulcus and middle temporal gyrus: a key component of the semantic system in the human brain. J Comp Neurol. (2023) 531:1987–95. doi: 10.1002/cne.25521, PMID: 37434287

[36] Apkarian AV Bushnell MC Treede RD Zubieta JK. Human brain mechanisms of pain perception and regulation in health and disease. Eur J Pain. (2005) 9:463–84. doi: 10.1016/j.ejpain.2004.11.001, PMID: 15979027

[37] Zhang J Su J Wang M Zhao Y Zhang QT Yao Q. The sensorimotor network dysfunction in migraineurs without aura: a resting-state fMRI study. J Neurol. (2017) 264:654–63. doi: 10.1007/s00415-017-8404-4, PMID: 28154971

[38] Zou Y Tang W Qiao X Li J. Aberrant modulations of static functional connectivity and dynamic functional network connectivity in chronic migraine. Quant Imaging Med Surg. (2021) 11:2253–64. doi: 10.21037/qims-20-588, PMID: 34079699 PMC8107335

[39] Coppieters I Meeus M Kregel J Caeyenberghs K de Pauw R Goubert D. Relations between brain alterations and clinical pain measures in chronic musculoskeletal pain: a systematic review. J Pain. (2016) 17:949–62. doi: 10.1016/j.jpain.2016.04.005, PMID: 27263992

[40] Jellinger KA. Neuroscience in medicine. New York, NY: Humana Press (2008).

[41] Xie YF Huo FQ Tang JS. Cerebral cortex modulation of pain. Acta Pharmacol Sin. (2009) 30:31–41. doi: 10.1038/aps.2008.14, PMID: 19079295 PMC4006538

[42] Seifert F Maihöfner C. Functional and structural imaging of pain-induced neuroplasticity. Curr Opin Anaesthesiol. (2011) 24:515–23. doi: 10.1097/ACO.0b013e32834a1079, PMID: 21822136

[43] Quintero GC. Advances in cortical modulation of pain. J Pain Res. (2013) 6:713–725. doi: 10.2147/JPR.S45958, PMID: 24092997 PMC3788691

[44] Peters ML. Emotional and cognitive influences on pain experience. Mod Trends Pharmacopsychiatry. (2015) 30:138–52. doi: 10.1159/000435938 26436897

